# Supplementary material for: Hippocampal sequences represent working memory and implicit timing
Source: bioRxiv. 2025 Mar 17:2025.03.17.643736. Preprint. [Version 1] doi: 10.1101/2025.03.17.643736 (PMC11956965; doi:10.1101/2025.03.17.643736)
Supplement: 1 [file NIHPP2025.03.17.643736V1-supplement-1.pdf]

bioRxiv preprint doi: https://doi.org/10.1101/2025.03.17.643736; this version posted March 17, 2025. The copyright holder for this preprint (which was not certified by peer review) is the author/funder, who has granted bioRxiv a license to display the preprint in perpetuity. It is made available under aCC-BY 4.0 International license.

SUPPLEMENTAL FIGURES

bioRxiv preprint doi: https://doi.org/10.1101/2025.03.17.643736; this version posted March 17, 2025. The copyright holder for this preprint (which was not certified by peer review) is the author/funder, who has granted bioRxiv a license to display the preprint in perpetuity. It is made available under aCC-BY 4.0 International license.

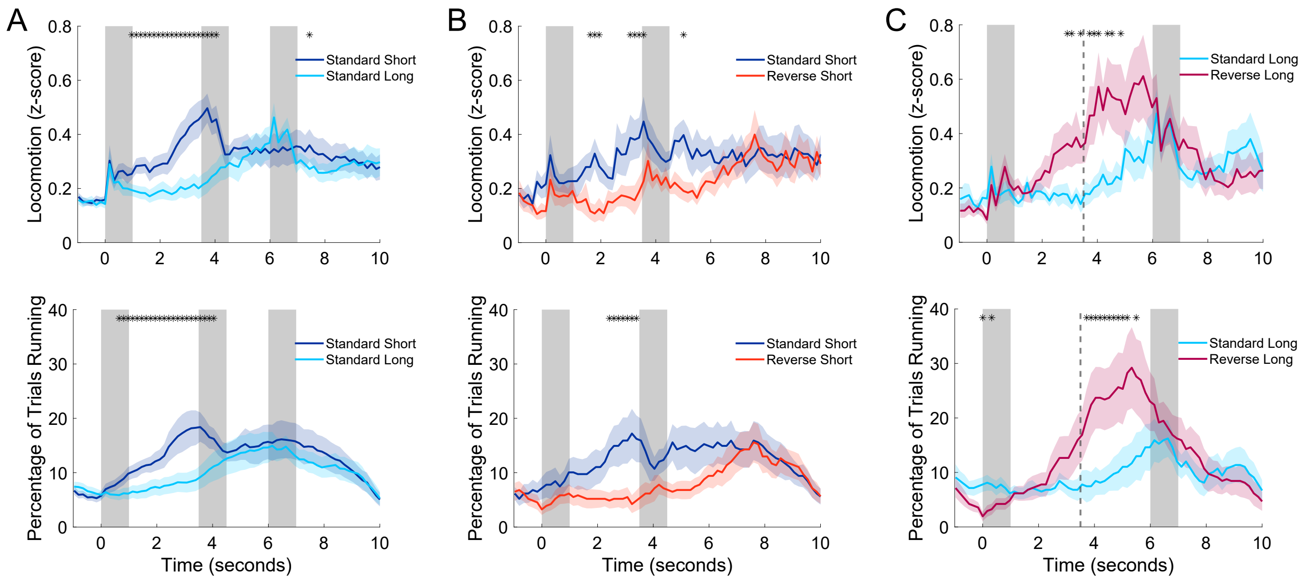

Supplemental Figure 1: Running differences during delays showed evidence of implicit timing. A) Top panel: locomotion is z-scored voltage signal from motion sensor binned to 1/6 second. Bottom panel: percentage of trials with running in a given bin. For both panels, comparison was made between all standard short and standard long trials from the last 2 standard days and first 2 reverse days. The gray bar at time point 0 is the 1st odor for both trial types, while the one at 3.5 is the 2nd odor for standard-short trials and the one at 6 is the 2nd odor for standard-long trials. Thick lines represent the mean of 44 recording sessions (11 mice across 4 days), and shaded area represents standard error of the mean. Asterisks represent bins of 1/6 second that were significantly different (Two-Way ANOVA animal and day, corrected for multiple comparisons with Benjamini-Hochberg procedure, $p < 0.01$). **B)** Same as (A), but comparison between standard-short and reverse-short trials from first 2 reverse days with the number of trials balanced by taking the nearest neighbor standard trial for each reverse trial. Statistics were the same as (A), but for only 22 recording sessions. **C)** Same as (B), but comparison between standard-long and reverse-long trials. The dotted line represents the expected 2nd odor arrival on reverse-long trials.

bioRxiv preprint doi: https://doi.org/10.1101/2025.03.17.643736; this version posted March 17, 2025. The copyright holder for this preprint (which was not certified by peer review) is the author/funder, who has granted bioRxiv a license to display the preprint in perpetuity. It is made available under aCC-BY 4.0 International license.

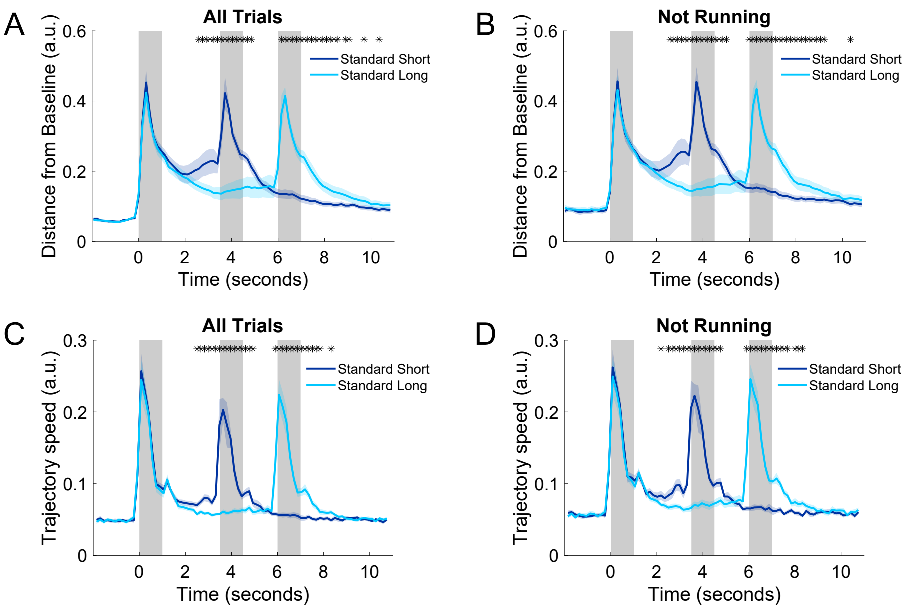

Supplemental Figure 2: Locomotion running patterns did not explain effects of neural trajectories. A) Same as Figure 3 B-C but comparing standard short and standard long trials. **B)** Same as (A) but excluding trials in which a 1-second period of running occurred during the delay period. **C)** Same as Figure 3 D-E but comparing standard short and standard long trials. **D)** Same as (C) but excluding running trials.
